# Application of BisANS fluorescent dye for developing a novel protein assay

**DOI:** 10.1371/journal.pone.0215863

**Published:** 2019-04-19

**Authors:** Zsolt Datki, Zita Olah, Lilla Macsai, Magdolna Pakaski, Bence Galik, Gabor Mihaly, Janos Kalman

**Affiliations:** 1 Department of Psychiatry, Faculty of Medicine, University of Szeged, Szeged, Hungary; 2 Department of Clinical Molecular Biology, Medical University of Bialystok, Bialystok, Poland; University of California, Davis, UNITED STATES

## Abstract

In many biology- and chemistry-related research fields and experiments the quantification of the peptide and/or protein concentration in samples are essential. Every research environment has unique requirements, e.g. metal ions, incubation times, photostability, pH, protease inhibitors, chelators, detergents, etc. A new protein assay may be adequate in different experiments beyond or instead of the well-known standard protocols (e.g. Qubit, Bradford or bicinchoninic acid) in related conceptions. Based on our previous studies, we developed a novel protein assay applying the 4,4′-Dianilino-1,1′-binaphthyl-5,5′-disulfonic acid dipotassium salt (BisANS) fluorescent dye. This molecule has several advantageous properties related to protein detection: good solubility in water, high photostability at adequate pH, quick interaction kinetics (within seconds) with proteins and no exclusionary sensitivity to the chelator, detergent and inhibitor ingredients. The protocol described in this work is highly sensitive in a large spectrum to detect protein (100-fold diluted samples) concentrations (from 0.28 up to more than 100 μg/mL). The BisANS protein assay is valid and applicable for quantification of the amount of protein in different biological and/or chemical samples.

## Introduction

Accurate peptide and/or protein quantification is essential in a multitude of research topics. Different methods were developed to measure the amount of proteins originating from various types of biological samples. A majority of them are fluorescence- (e.g. Qubit) or absorbance-based assays, such as the traditional Coomassie blue G-250 dye-binding [1] (Bradford) and the bicinchoninic acid (BCA) [2] assay. Both the Bradford and the BCA assays are based on color change in the visible spectrum as a response to the presence of proteins. The color formation observed in the Bradford assay is a result of complex formation between proteins and the Coomassie blue G-250 dye through electrostatic and hydrophobic interactions, where the anionic blue form of the dye is stabilized and could be measured [3]. The BCA assay is based on the reduction of Cu^2+^ to Cu^1+^ by protein in an alkaline medium. Cu^1+^ forms a complex with BCA, resulting a colored water-soluble chelate [2]. The intensity of the color change by these assays is measured by absorbance photometry at 595 nm and 562 nm for the Bradford and BCA assays respectively [4]. Both methods allow the detection of proteins in μg/mL range. However, every assay has its own specific limitation and unique requirements (different incubation times, stabilizations, metal ions, pH, photosensitivity, chelator- and detergent sensitivity) [5].

Several different fluorescent dyes are capable of measuring total protein content like 3-(4-carboxybenzoyl)quinoline-2-carboxaldehyde (CBQCA). Moreover, the CBQCA is well suited for accurate quantitation in the presence of lipids, membrane fractions and for lipoproteins and small peptides [6]. Based on their applications and high sensitivity standardized assays are commercially available. Other fluorescent dyes, such as 8-Anilino-1-naphthalenesulfonic acid and its dimeric analogue 4,4′-Dianilino-1,1′-binaphthyl-5,5′-disulfonic acid dipotassium salt (BisANS) are applied in various fields of protein analysis e.g. to assess surface hydrophobicity [7]; to probe active sites of enzymes [8]; to monitor unfolding and refolding processes [9]; to characterize neurodegenerative-related protein aggregates formation [10–11] or fibrillation [12] and to monitor tubulin assembly [13]. Fu et al. (2005) [14] revealed a chaperone-like activity for BisANS in preventing protein aggregation and in partially attenuating the heat-inactivation of enzymes. In our previous study we described a novel application of BisANS, which is capable of labelling damaged live/degenerated neurons and neuroblastoma cells [15]. Additionally, we used *in vivo* detection of exogenic peptide aggregates (e.g. beta-amyloid) in an invertebrate bdelloid rotifer model [16].

The fluorescence properties of BisANS strongly depend on its interaction with protein molecules similar to other protein specific dyes [17], causing changes of polarity and viscosity of the environment [18]. This non-covalent dye binds to non-specifically at multiple sites of many proteins [19] through its hydrophobic and electrostatic interactions [18]. The main advantages of BisANS are the high fluorescent intensity and great sensitivity, since it lacks an aspecific background resulted by different wavelength ranges of excitation (380–410 nm) and emission (510–530 nm) [15]. All these characteristics of the dye enable the application of the BisANS in a greater variety of protein research.

Thus, our aim was to develop a novel, fluorescence-based protein quantification method with applying BisANS dye in absence or in presence of chelator, detergent and protease inhibitors.

## Material and methods

### Protein preparations

**Fig 1:**
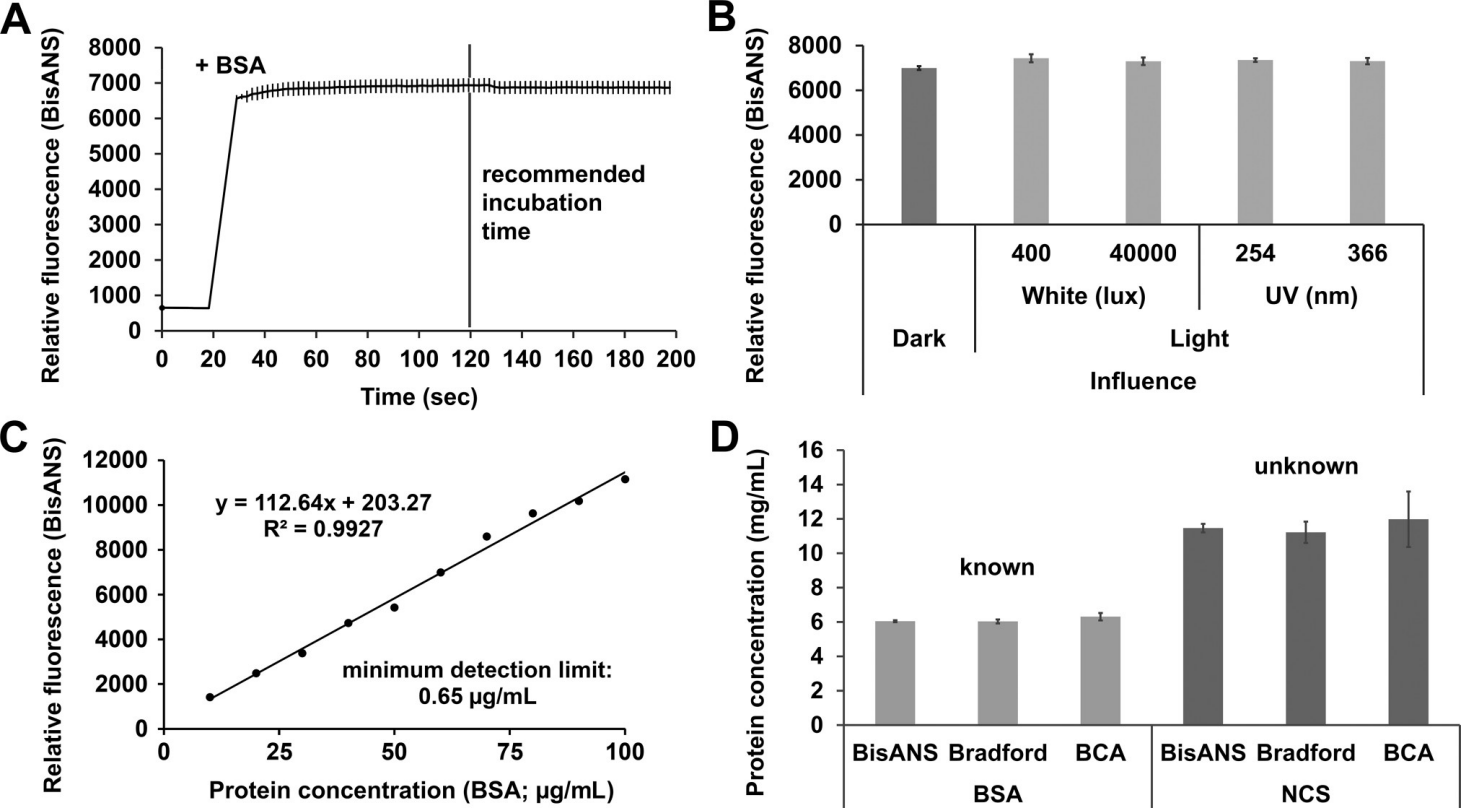
Characterization, application and validation of BisANS assay in basic medium. (A) Saturation curve of fluorescent signal after BisANS–BSA interaction. (B) Stability and protein binding capability of BisANS after various light influences. (C) Calibration curve formed by gradual BSA doses detected by BisANS. (D) Application and validation of BisANS-based assay to measure known (BSA) and unknown (NCS) protein concentrations. The mean is presented and the error bars show the S.E.M. For statistical analysis, one-way ANOVA was used followed by the Bonferroni *post hoc* test.

**Fig 2:**
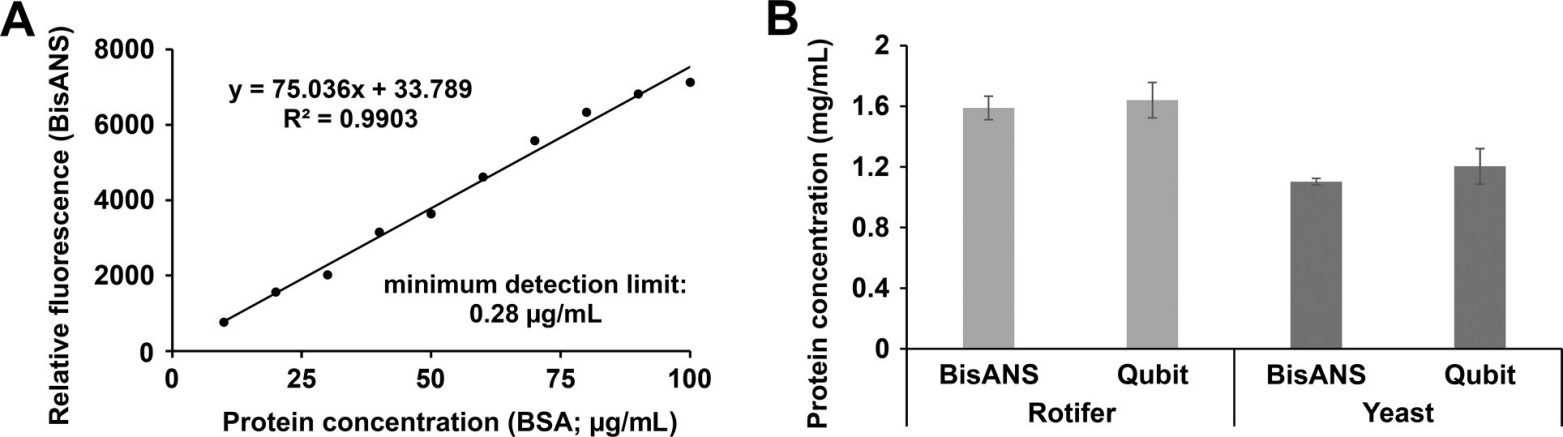
Application and validation of the BisANS assay on protein samples prepared and isolated in lysis buffer. (A) Calibration curve formed by gradual BSA doses (dissolved in lysis buffer) detected by BisANS. (B) Application of BisANS assay simultaneously with Qubit assay on isolated protein samples from a bdelloid rotifer or yeast. The means are presented by the columns and the error bars show the S.E.M. For statistical analysis, one-way ANOVA was used followed by the Bonferroni *post hoc* test.

In order to validate our novel protein assay, different protein samples were used. The samples and the BisANS dye (Sigma-Aldrich, MO, USA) were dissolved in distilled water (DW; Millipore type, ultra-pure, demineralized) based calcium and magnesium free basic medium (content in mM: NaCl 115, KCl 3, HEPES 25, D-Glucose 10, pH 6.5; ingredients were obtained from Sigma-Aldrich, MO, USA). We tested a pure protein (bovine serum albumin, BSA; Sigma-Aldrich, MO, USA) and a complex blood sample without isolation (newborn calf serum; NCS; Sigma-Aldrich, MO, USA; Fig 1) or samples derived from a bdelloid rotifer and yeast. They were isolated in lysis buffer, where the basic medium was supplemented with the chelator ethylenediaminetetraacetic acid (EDTA, 2 mM; Sigma-Aldrich, MO, USA), the detergent sodium dodecyl sulfate (SDS, 0.2%; Aldrich, MO, USA) and inhibitors (10 μM/mL leupeptin hydrochloride and 1 μg/mL pepstatin A; Sigma-Aldrich, MO, USA) in order to test the tolerance of our assay (Fig 2). The source of protein was the bdelloid rotifer and yeast samples. The isolation steps were the following: the intact animals or the yeast cells were centrifuged at 3000 x g, at room temperature for 10 minutes. The supernatants were discarded, and the pellets were resuspended in lysis buffer (2x10^5^ animals/ mL or 10^6^ yeast cells/mL). The samples were frozen to -75°C for 60 minutes. After thawing, they were ultrasonicated (45 Hz, Emmi-40 HC, EMAG AG, Mörfelden-Walldorf, Germany) for 10 minutes. The isolates were centrifuged at 1500 x g for 5 minutes to eliminate the exoskeletons or cell debris from the homogenates. The BSA stock solution (10 mg/mL) was diluted by basic medium (Fig 1) or lysis buffer (Fig 2) to set up the calibration curves. The concentrations were from 1 to 10 mg/mL, with 1 respective unit steps (n = 3 wells per each dose, triplicated). According to the current ethical regulations, no specific ethical permission was needed. Our experiments were carried out in accordance with globally accepted norms: Animals (Scientific Procedures) Act, 1986, associated guidelines, EU Directive 2010/63/EU for animal experiments, and the National Institutes of Health guide for the care and use of Laboratory animals (NIH Publications No. 8023, revised 1978). Animal studies comply with the ARRIVE guidelines. The BSA solutions were ultrasonicated for 10 minutes to dissolve the coagulations. In all protein assays the respective medium was used as a blank and the BisANS in itself served as the unspecific background. In all cases 0.5 μL of protein samples were added to the each well where the working volume was 50 μL/well (1:100 dilution). Measurements were carried out in a 96-well microplate with half-area (Cornig Inc., NY, USA).

### BisANS assay

The BisANS was diluted in basic medium (pH 6.5), the working concentration was 50 μM, using BMG NOVOstar plate reader (BMG Labtech, Ortenberg, Germany). The extinction/emission was 405/520 nm and the number of flash per well and cycle was 30. Orbital shaking was applied before the first cycle, the shaking time was 3 sec and the plate-rounds per minutes were 600. The gain adjustment was set to both medium and dye background. The medium readings were normalized to the dye background, and they were averaged with the dye readings. During the saturation measurement (Fig 1A) and the light influence tests (Fig 1B) 6 mg/mL BSA stock solution was used (n = 18 wells, respectively). The saturation was recorded for 200 sec. After 20 sec background recording, 0.5 μL BSA stock solution was added automatically by the plate reader-inserted pipettor and the final volume of protein-dye content was 50 μL per well. To determine the light stability of BisANS, different aliquots (50 μM) in 0.2 mL PCR tubes (Thermo Fisher Scientific, MA, USA) were illuminated by various intensity of light for five minutes before the measurements. We tested white (400 and 40,000 lux) and UV light (254 and 366 nm). Based on the results of saturation experiments, 6 mg/mL BSA solution was measured after 2 minutes at room temperature incubation in darkness. The light stability measurements were recorded and calculated similarly to the elements of the calibration curve (Fig 1C). To validate our assay 6 mg/mL BSA solution (n = 12 wells) and NCS (n = 12 wells) were determined by BisANS assay parallel with Bradford and BCA assays (Fig 1D).

### Bradford assay

The commercially available Bradford reagent (Sigma-Aldrich, MO, USA) was used in 1:5 dilution with basic medium (Fig 1D). The plate was incubated in darkness for 30 minutes; the colorimetric change was detected by microplate reader (SpectraMax Plus 384, Molecular Devices, LLC., CA, USA) set at 592 nm. The readings were normalized to the Bradford reagent background.

### BCA protein assay

The commercially obtainable BCA Protein Assay kit [20] (Merck & Co., Inc., NJ, USA) was used (Fig 1D). The protocol given by the manufacturer was followed. Briefly, 4% cupric sulphate was diluted by 20-fold in BCA Solution (BCA, sodium carbonate, sodium tartrate, and sodium bicarbonate with 0.1 M NaOH, pH 11.25) as working solution. The plate was incubated for 30 minutes at 37°C in darkness. The absorbance was measured by microplate reader (SpectraMax Plus 384, Molecular Devices, LLC., CA, USA) set at 562 nm. The standardized results were normalized to the working solution background.

### Qubit assay

The commercially available Qubit Assay kit [21] (Thermo Fisher Scientific, MA, USA) was used (Fig 2B). The protocol given by the manufacturer was applied (n = 12, PCR Eppendorf tubes). Briefly, 1 μL dye was diluted in 199 μL buffer, then the 1 μL rotifer or yeast isolates were added to 199 μL mix. The reaction mix was incubated in dark for 15 minutes and the readings were obtained by the Qubit Fluorometer 2.0 (Thermo Fisher Scientific, MA, USA). The final concentration of the samples was calculated by its own reader.

### Statistics

All raw data is provided as supplementary information (S1 Dataset). We presented the mean, and the error bars showed the standard error of the mean (S.E.M.). The one-way ANOVA was used for the comparison, followed by the Bonferroni *post hoc* test with GraphPad Prism 7.0b software (GraphPad Software Inc., CA, USA).

## Results and discussion

Different methods are used to quantify the total amount of protein in a multitude of research topics; however, each assay has its own limitations (e.g. sensitivity to chelators and detergents). Due to the development of high throughput proteomic screening, there is an increasing demand for developing new, easy-to-use approaches. The BisANS fluorescent dye has been introduced in several fields of protein analysis. To characterize our novel BisANS-based protein quantification assay we tested it with protein samples prepared in basic medium (Fig 1) or in lysis buffer (Fig 2). First of all, we recommended an optimal incubation time and environment for this assay using BSA as standard in chelator, detergent and inhibitor free basic medium. To reach the highest reliability, the relative fluorescence intensity, and the recommended incubation time were indicated (Fig 1A). We suggest subjectively 120 sec incubation time for saturation of fluorescent signal; however, we believe that it is unnecessary to wait any longer. The photostability of the BisANS was tested under different light conditions: dark, white (400 and 40,000 lux) and ultraviolet (UV; 254 and 366 nm) ones (Fig 1B). No significant changes were observed. The concentration range and minimum detection limit were evaluated by BSA standard ranging from 0 to 100 μg/mL, and the correlation coefficient (R^2^ = 0.9927; y = 112.64x + 203.27) was calculated (Fig 1C). The minimum detection limit was 0.65 μg/mL. To validate our assay, Bradford and BCA methods were used, paralelly measuring known (BSA) and unknown (NCS) protein concentrations. We found that the BisANS assay proved to be the most accurate (Fig 1D).

To test the compatibility of our BisANS assay with interfering agents, we supplemented the basic medium with chelator (EDTA), detergent (SDS) and protease inhibitors such as leupeptin and pepstatin A, thus forming a lysis buffer. The calibration curve was well-fitted in this environment (R^2^ = 0.9903; y = 75.036x + 33.789; Fig 2A). The absolute fluorescence intensities decreased approximately by 35% compared to the corresponding data measured in the basic medium. The minimum detection limit was 0.28 μg/mL. Since the previously applied Bradford and BCA methods are sensitive to the additional ingredients (EDTA, SDS and inhibitors), Qubit assay was used to validate our readings. The protein samples originated from living organisms, such as a bdelloid rotifer *(Philodina acuticornis)* and yeast *(Saccharomyces cerevisiae)*. These biological samples were isolated in the lysis buffer and their protein contents were measured both with Qubit and BisANS (Fig 2B). No significant difference was detected between the data measured by our assay and the Qubit.

In our experiments, we were the first to present the new application of the BisANS dye, as a basis of total protein quantification method besides or instead of another equivalent technique. This dye has high photostability; therefore, no special care during incubation is necessary (unlike in the case of other fluorescent dyes, e.g. CBQCA) [6]. We demonstrated the advantages of BisANS-based assay, namely: good solubility in water, short incubation time, high protein affinity and no exclusionary sensitivity to EDTA, SDS and protease inhibitors applied in our experimental settings with optimized concentrations; reliability and repeatability in broad spectrum of protein content with very low minimal and relatively high maximal detection limit in comparison to other analog methods [1,2,21,22,23].

In summary, we have successfully developed and characterized a novel, BisANS-based total protein quantification method (S1 Graphical Abstract**)**, with numerous advantages, which could be useful in many areas of protein science.

## Supporting information

S1 Dataset Raw data. All raw data from the study is provided in the RAW data.zip supplementary information.
(ZIP)

S1 Graphical Abstract The BisANS has advantageous properties in protein detection: high photostability, quick interaction kinetics; no sensitivity to chelator, detergent and inhibitors. (TIF)
