# Supplementary figures and images for: Application of BisANS fluorescent dye for developing a novel protein assay
Source: PLoS One. 2019 Apr 19;14(4):e0215863. doi: 10.1371/journal.pone.0215863 (PMC6474611; doi:10.1371/journal.pone.0215863)

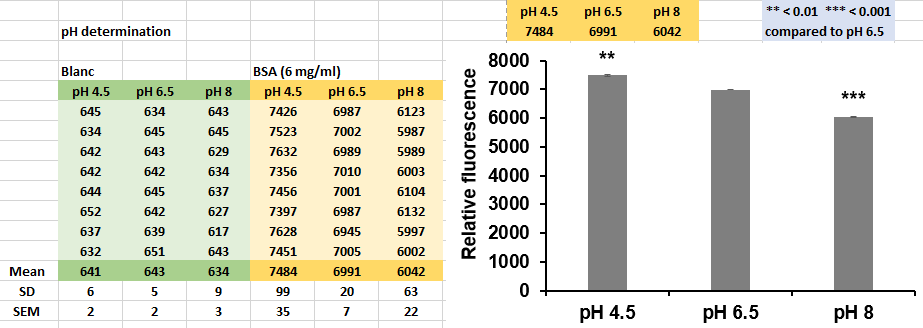

Supplement: S1 Dataset — All raw data from the study is provided in the RAW data.zip supplementary information. (ZIP) [file pone.0215863.s001.zip › RAW data/pH determination.tif]

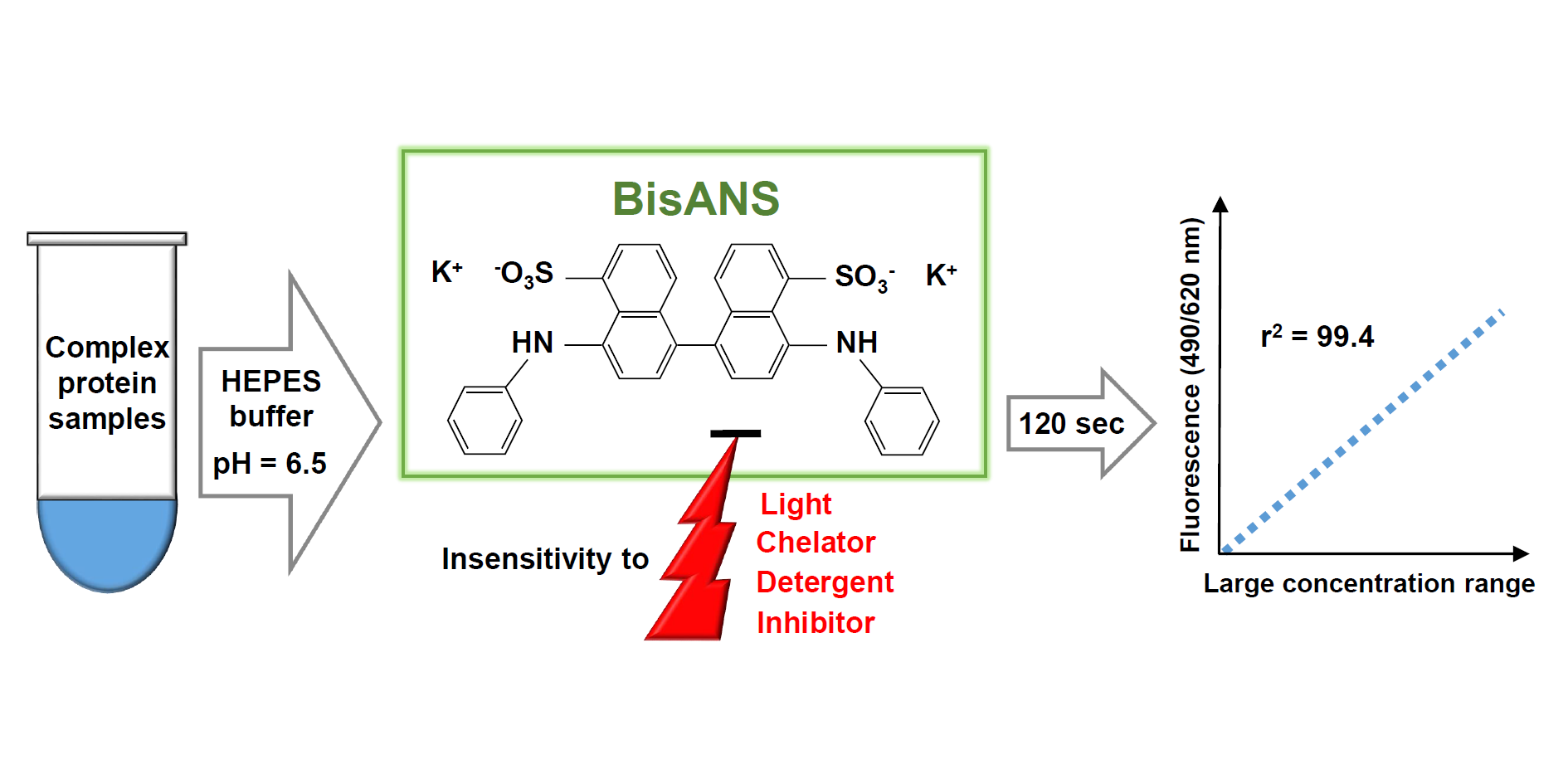

Supplement: S1 Graphical Abstract — (TIF) [file pone.0215863.s002.tif]
